# β‑Cyclodextrin/Nerolidol Inclusion Complex as a Potential Ecofriendly Antifungal System against *Botrytis cinerea* in Postharvest Strawberries

**DOI:** 10.1021/acsomega.6c06824

**Published:** 2026-09-26

**Authors:** Aldrey N. R. Corrêa, Lara E. Zanotelli, Jessica F. Hoffmann, Adriano Brandelli

**Affiliations:** † Department of Food Science, 28124 Universidade Federal do Rio Grande do Sul, 91501-970 Porto Alegre, Brazil; ‡ Technological Institute in Food for Health, 37906 Universidade do Vale do Rio dos Sinos, 93022-750 São Leopoldo, Brazil; § Center of Nanoscience and Nanotechnology, Universidade Federal do Rio Grande do Sul, 91501-970 Porto Alegre, Brazil

## Abstract

β-Cyclodextrin (β-CD) encapsulation may offer a strategic approach to overcome the high volatility of nerolidol (NE) for controlling *Botrytis cinerea* decay in postharvest strawberries. In this study, a β-cyclodextrin/nerolidol inclusion complex (β-CD/NE) was prepared by freeze-drying to improve the stability and performance of NE. The complex exhibited high complexation efficiency (96.71%) and nanoscale particle size (484.9 nm). FTIR, XRD, and thermal analyses evidenced strong molecular interactions and structural modifications, and an improvement in the thermal stability of the sesquiterpene, while solid-state ^13^C NMR and SAXS indicates the formation of host–guest inclusion complex through the appearance of characteristic nerolidol resonances and structural modifications in the β-CD matrix. The β-CD/NE showed significantly higher antifungal activity against *B. cinerea* than free NE *in vitro*, with inhibition increasing from 17.9% to 65.8% between 200 and 6400 μM, while free NE reached a maximum inhibition of 43.6% at the same concentration range. Strawberries treated with β-CD/NE and stored at 5 °C for 10 days showed a visible decrease of fungal growth and a significant reduction in mass loss compared to untreated fruits, suggesting a high preservation potential. This study advances the application of natural antifungal systems, as the utilization of β-CD/NE inclusion complex as a postharvest antifungal system against *B. cinerea* in strawberries has not been reported elsewhere.

## Graphical abstract

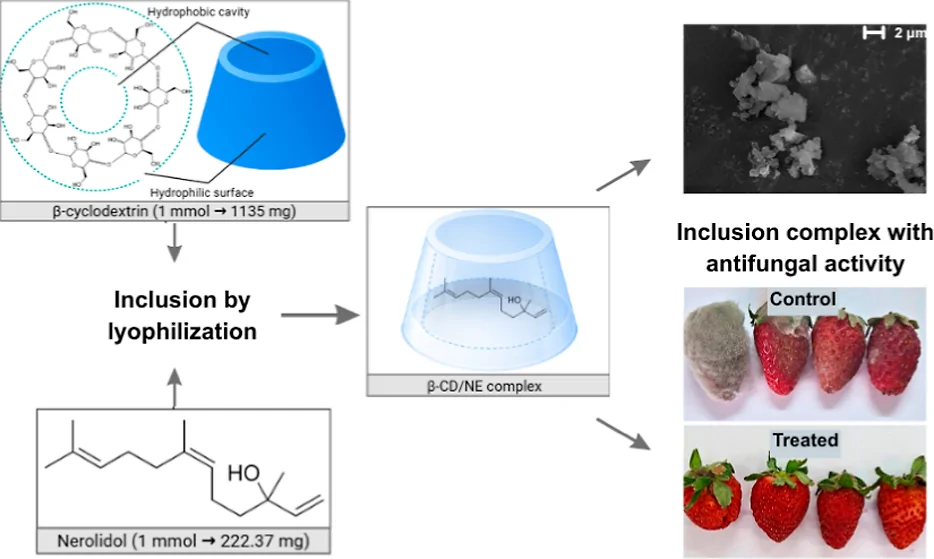

## 1 Introduction

*Botrytis cinerea* is a widespread necrotrophic fungal pathogen responsible for gray mold, one of the most destructive postharvest diseases affecting strawberries. This fungus can infect fruits during both pre- and postharvest stages, causing rapid tissue maceration, soft rot, and severe economic losses.^1,2^ Given that strawberry is an economically relevant crop worldwide, with an annual production value exceeding US$ 2.3 billion in the USA alone, the presence of gray mold represents the primary driver for fruit rejection across the entire supply chain. This fungus can infect fruits during both pre- and postharvest stages, causing rapid tissue maceration, soft rot, and severe financial losses to growers, shippers, and consumers.^1,3^ Under wet conditions, losses may exceed 80% if effective control measures are not applied.^3^ The pathogen displays a complex infection strategy involving enzymatic degradation, toxin secretion, and manipulation of host cell death, which contributes to its high virulence and adaptability.^1^ Although substantial efforts have been made, the development of strawberry cultivars resistant to *B. cinerea* has not been achieved, and current control strategies remain largely dependent on chemical fungicides.^3^

The intensive use of synthetic fungicides has raised concerns regarding environmental contamination, the emergence of resistant *B. cinerea* strains, and significant human health threats, such as skin and eye irritation, endocrine disruption, neurological and liver damage, and a higher risk of certain cancers.^4,5^ These health and safety concerns are specifically driven by the presence of harmful chemical drug residues remaining on the surface of fresh fruits, which heightens consumer concerns regarding food safety. Consequently, these toxicological and environmental limitations have motivated increasingly regulatory restrictions on the authorization and use of synthetic postharvest chemicals.^2,4^ Moreover, chemical disinfectants such as ethanol, acetic acid, and oxidizing agents may negatively affect fruit quality and pose additional environmental challenges.^5^ These limitations have motivated the search for safer and more sustainable alternatives to control gray mold and reduce postharvest losses in strawberries.^6–8^

Natural bioactive compounds, particularly essential oil constituents, have gained attention due to their antimicrobial and antifungal properties.^9^ Sesquiterpenoids represent an abundant class of plant-derived compounds with broad-spectrum antifungal activity.^9^ Nerolidol (NE), an acyclic sesquiterpene alcohol found in several essential oils, exhibits antifungal, antimicrobial, antioxidant, and anti-inflammatory effects and is classified as generally recognized as safe by Food and Drug Administration (FDA), which allows its use as a flavoring agent in food.^10–12^ However, its practical application is limited by low water solubility, high volatility, and sensitivity to light and oxidation.^10,13^

Encapsulation using cyclodextrins has been explored to overcome these limitations, once these cyclic oligosaccharides form inclusion complexes with hydrophobic compounds, improving their solubility, stability, and controlled release.^14–16^ Previous studies have shown that β-cyclodextrin (β-CD) complexes containing essential oil derivatives can effectively inhibit *B. cinerea*, particularly when incorporated into active packaging materials.^4,17^ NE has also been successfully encapsulated in β-CD-based systems, such as cyclodextrin-liposome complexes that enhanced its photostability and prolonged its release.^10^ Recent structural studies have further confirmed the formation of stable β-CD/nerolidol inclusion complex (β-CD/NE), revealing specific host–guest interactions between the terpene molecule and the cyclodextrin cavity.^18^

However, only a few studies describe the potential application of β-CD-NE complexes in pharmaceutical or food model systems.^10,19,20^ Although the antifungal activity of NE against *B. cinerea* has been reported, the antifungal effect of β-CD/NE and its direct application as a postharvest treatment for fresh produce has not yet been investigated. In this regard, the hypothesis of this study is that β-CD/NE inclusion complex can be an effective antifungal system against *B. cinerea* in postharvest strawberries. In this work, the β-CD/NE complex was prepared, characterized, and evaluated for its antifungal efficacy against *B. cinerea* under *in vitro* conditions and on strawberry fruits. This approach advances the development of safe, natural, and effective strategies based on natural preservatives for reducing postharvest losses and extending the shelf life of strawberries.

## 2 Experimental Section

### 2.1 Chemicals

Nerolidol (NE; 98% purity, mixture of cis and trans), β-cyclodextrin (β-CD; ≥97% purity) were purchased from Sigma-Aldrich (St. Louis, MO, USA). Potato-Dextrose agar (PDA) was obtained from Merck (Darmstadt, Germany). Capric/caprylic triglyceride was provided by Delaware (Porto Alegre, Brazil). Polysorbate 80 (Tween 80) was acquired from Synth (Diadema, Brazil). Ultrapure water was obtained using a Milli-Q purifier (Millipore, Burlington, MA, USA). All other reagents were of analytical grade, and ultrapure water was used to prepare all solutions.

### 2.2 Preparation of the β-CD/NE Inclusion Complex

The β-CD/NE was prepared by a freeze-drying method.^11^ Initially, NE (C_15_H_26_O; molecular weight = 222.37 g/mol) and β-CD (C_42_H_70_O_35_; molecular weight = 1134.98 g/mol) were mixed at an equimolar ratio (1:1), calculated based on their molecular weights, and dispersed in 20 mL of ultrapure water. The mixture was maintained under magnetic stirring at 400 rpm for 36 h at room temperature, in a 50 mL beaker covered with a watch glass, to facilitate molecular interaction and inclusion complex formation (Figure 1). Subsequently, the suspension was frozen at −80 °C for 30 min and lyophilized under vacuum conditions (pressure ≤0.1 mbar, condenser temperature at −50 °C) for 4 days to ensure complete sublimation of ice and removal of residual moisture, yielding β-CD/NE as a dry powder. For comparison purposes, a β-CD inclusion complex containing capric/caprylic triglyceride was prepared following the same procedure and used as a control (β-CD/C).

**Figure 1:**
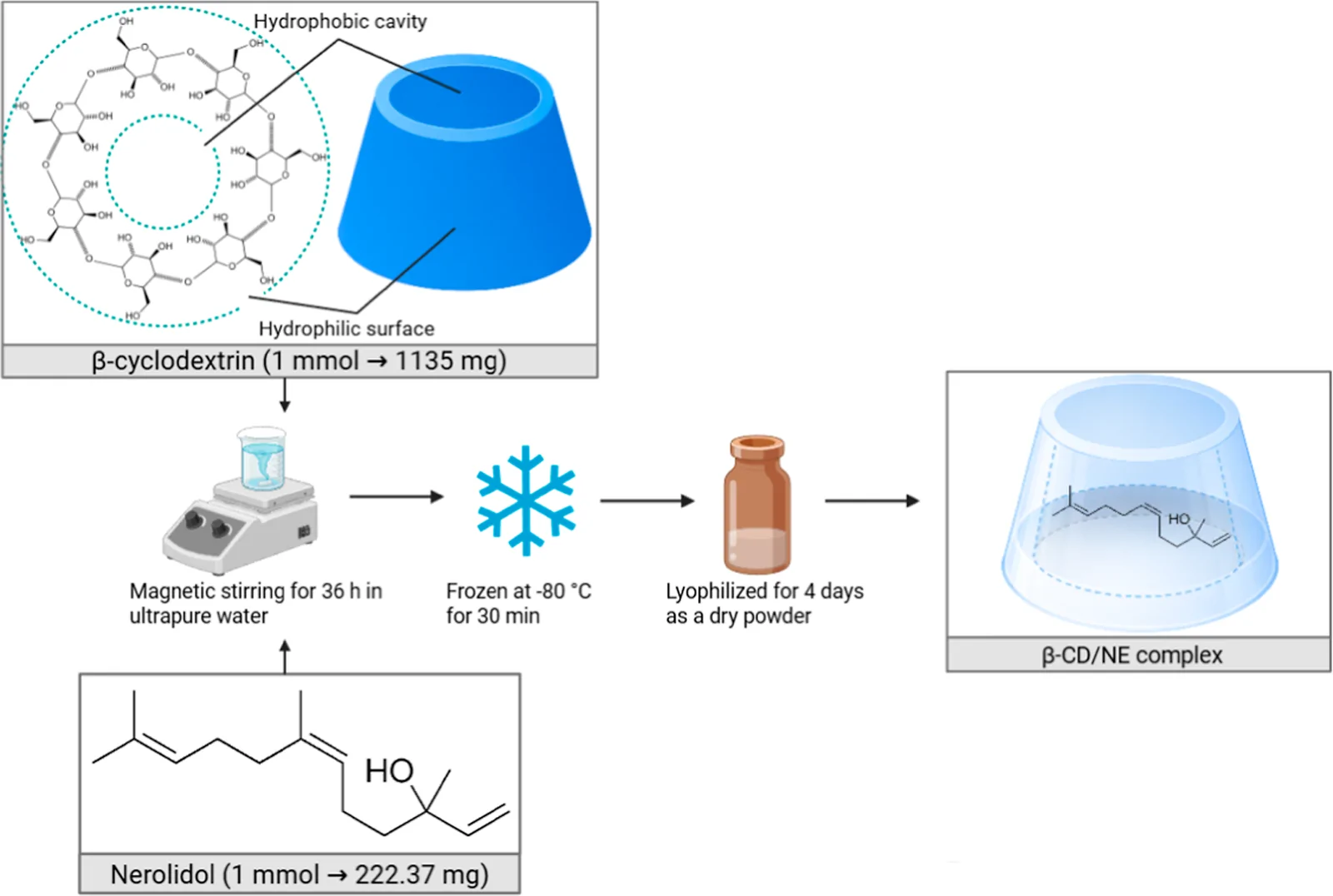
β-CD/NE inclusion complex formation (created with BioRender.com).

### 2.3 Complexation Efficiency

#### 2.3.1 Determination of Nerolidol Concentration in β-CD/NE Aqueous Solution

The complexation efficiency (CE %) of the β-CD/NE was determined by quantifying the amount of noncomplexed NE using a high-performance liquid chromatography (HPLC) protocol.^19^ Initially, 5 mg of the β-CD/NE complex was dispersed in 1 mL ultrapure water by vortexing for 30 s followed by ultrasonication using a probe-type ultrasound device (Unique OF S500, Americana, Brazil) equipped with a 3 mm titanium probe at 50 kHz and 250 W for 1 min until complete dispersion was achieved. The resulting suspension was subjected to centrifugal filtration (15,600*g* for 1 h at 4 °C) using a membrane filter (Ultracel-3k, Merck Millipore Ltd., Tullagreen, Ireland) with a molecular weight cutoff (MWCO) of 3 kDa, thereby separating free NE from the β-CD/NE inclusion complexes.

After centrifugation, the filtrate containing free NE was collected and diluted with HPLC-grade methanol to ensure adequate solubilization and compatibility with the chromatographic conditions. Analyses were performed using a Shimadzu high-performance liquid chromatography system (Shimadzu Corporation, Kyoto, Japan) equipped with a CBM-20A system controller, LC-20AT pump, DGU-20A SR online degasser, SIL-20A autosampler, CTO-20A column oven, and SPD-M20A photodiode array (PDA) detector. Quantification of NE was carried out using a Zorbax Eclipse Plus C18 column (250 × 4.6 mm, 5 μm) under isocratic conditions with water/methanol (50:50, v/v) as the mobile phase at a flow rate of 1.0 mL min^–1^. The column temperature was maintained at 25 °C, the injection volume was 20 μL, and the total run time was 10 min. Detection was performed at 212 nm. Quantification was based on an external calibration curve prepared in the concentration range of 10–150 μg mL^–1^. The CE % was calculated according to the eq 1.

1 $$CE\;\%=\frac{NE\;amount\;complexed}{NE\;amount\;initially\;added}\times 100$$

where NE amount complexed corresponds to the difference between the initial NE amount and the free NE quantified in the filtrate after separation.

#### 2.3.2 Method Validation

The HPLC method was validated according to the Brazilian Health Regulatory Agency (ANVISA) Resolution RDC No. 166/2017. Linearity was evaluated using six concentration levels ranging from 10 to 100 μg mL^–1^, with three replicate injections at each level. Calibration curves were constructed by plotting peak area versus analyte concentration using linear regression (*R* ^2^ = 0.9924). Representative chromatograms of the standard and the sample are presented in Figure S1.

Method precision (repeatability) was assessed by three consecutive injections of each concentration level under the same analytical conditions and expressed as the relative standard deviation (RSD). RSD values were below 5% for all evaluated concentrations. The limit of detection (LOD) and limit of quantification (LOQ) were determined experimentally based on signal-to-noise (S/N) ratios of approximately 3:1 and 10:1, respectively. The LOD and LOQ were established as 3 μg mL^–1^ and 10 μg mL^–1^, respectively.

### 2.4 Dynamic Light Scattering (DLS) and Zeta Potential Analysis

The intensity-weighted *Z*-average hydrodynamic diameter, particle diameter dispersity (D̵), and zeta potential of the β-CD/NE were determined using a Litesizer DLS 700 instrument (Anton Paar, Graz, Austria). The samples were dispersed in ultrapure water (1:100) and analyzed at 21 °C. Particle size measurements were performed by dynamic light scattering in side-scatter mode using quartz cuvettes, while zeta potential measurements were conducted by electrophoretic light scattering using omega-type capillary cuvettes. Water was used as the dispersant medium, and data were analyzed using the cumulant method.

### 2.5 Scanning Electron Microscopy (SEM)

The dried powder samples were deposited onto aluminum stubs using conductive carbon tape, and excess material was removed to avoid particle agglomeration. Prior to analysis, the samples were sputter-coated with a conductive layer of Au/Pd. SEM images were acquired using a field-emission scanning electron microscope (Zeiss, Oberkochen, Germany) operated at an accelerating voltage of 10.0 kV, with a working distance of approximately 7.5 mm and secondary electron detection. Micrographs were recorded at a magnification of 10,000×.

### 2.6 Fourier-Transform Infrared Spectroscopy (FTIR)

The spectra were recorded using a Spectrum Two FTIR spectrometer (PerkinElmer, Waltham, MA, USA). Data acquisition and processing were performed using Spectrum software (version 10.7.2, PerkinElmer). Spectra were collected in the range of 4000 to 500 cm^–1^ with a spectral resolution of 4 cm^–1^.

### 2.7 X-ray Diffraction (XRD)

XRD analyses were performed using a Rigaku diffractometer (Tokyo, Japan) equipped with an Ultima IV goniometer and a fixed monochromator (U4). Diffraction patterns of β-CD and the β-CD/NE inclusion complex were recorded in continuous-scanning mode using Cu Kα radiation at 40 kV and 20 mA. Data were collected in the 2θ range of 5–50°, with a step size of 0.02°, using a θ–2θ scanning configuration. For graphical presentation, the diffractograms were normalized between 0 and 1, solely for comparative visualization purposes; however, all crystallinity indices (Xc %) calculations were performed using only the raw data, without any normalization, according to the eq 2.

2 $$Xc\left(\%\right)=\frac{A_{crist}}{A_{total}}\times 100$$

where *A*_crist_: area of the crystalline peaks, obtained by integrating the peaks after subtracting the baseline; *A*_total_: total area of the diffractogram, corresponding to the sum of the crystalline and amorphous contributions, obtained by integrating the raw signal over the analyzed 2θ interval.

### 2.8 Small-Angle X-ray Scattering (SAXS)

SAXS measurements were performed using a Nano-inXider instrument (Xenocs, Grenoble, France) operating in very high resolution (VHR) mode. Lyophilized powder samples were analyzed at room temperature. Two-dimensional scattering patterns were recorded and radially integrated using Foxtrot software (version 3.2.7, Xenocs) to obtain one-dimensional *I*(*q*) profiles. For each sample, 60 frames were acquired and averaged to improve signal-to-noise ratio.

Background subtraction was performed using the corresponding empty holder measurement. The scattering intensity was analyzed as a function of the scattering vector *q* (Å^–1^). Data treatment and fitting were carried out using SasView software (version 6.1.2). The intermediate *q*-range (0.01–0.1 Å^–1^) was fitted using a power-law model, *I*(*q*) = *Aq* ^–*m*^, and the Porod exponent (*m*) was obtained from the slope of the log–log plot.

### 2.9 Nuclear Magnetic Resonance (NMR)

Solid-state ^13^C nuclear magnetic resonance (^13^C NMR) spectra were acquired using a 500 MHz spectrometer (11.74 T, Agilent Technologies, Santa Clara, CA, USA) equipped with a 4 mm MAS probe. Measurements were performed using the cross-polarization/magic-angle spinning (^13^C CP/MAS) technique. The resulting spectra were analyzed as chemical shift values (δ, ppm) to evaluate structural changes associated with complex formation.

### 2.10 Thermal Properties

Differential scanning calorimetry (DSC) analyses were performed using a DSC 8500 calorimeter (PerkinElmer, Waltham, MA, USA). Approximately 5 mg of each sample were accurately weighed into sealed aluminum pans and analyzed under a nitrogen atmosphere, with a purge gas flow rate of 10 mL/min. The samples were heated from 0 to 220 °C at a constant rate of 10 °C/min. After the heating cycle, the system was rapidly cooled to 20 °C.

Thermogravimetric analysis (TGA) of the composites was evaluated using a TGA analyzer (Q50; TA Instruments, New Castle, DE, USA). Samples were heated from 50 to 800 °C at 10 °C min^–1^ under nitrogen at 20 mL min^–1^.

### 2.11 Antimicrobial Activity by Agar Contact Method

The antifungal activity of the β-CD/NE against *B. cinerea* was evaluated using the agar contact method,^21^ and the results were directly compared with those obtained for free NE. Lyophilized β-CD/NE samples were initially dispersed in an aqueous solution containing 0.05% (v/v) Tween 80 and diluted to obtain the desired concentrations. The amounts of β-CD/NE required to reach final molar concentrations of 200, 400, 800, 1600, 3200, and 6400 μM were calculated based on the previously determined complexation efficiency (CE), ensuring equivalent molar concentrations of NE in all treatments. In parallel, free NE was prepared at the same molar concentrations (200–6400 μM) by dilution in 0.05% Tween 80 and used as a reference to compare antifungal activity.

The prepared solutions were homogenized into molten PDA and poured into sterile Petri dishes (90 mm in diameter). Each plate received 15 mL of the supplemented medium and was allowed to solidify at room temperature. A mycelial disc (3 mm in diameter), obtained from the actively growing margin of 7 day-old *B. cinerea* cultures, was placed at the center of each plate. The plates were incubated at 25 ± 2 °C for 7 days, and mycelial growth was measured along two perpendicular axes. Antifungal activity was expressed as percentage of inhibition (IP %), calculated at the end of the incubation period according to eq 3.

3 $$IP\;\%=\frac{C-T}{C}\times 100$$

where *C*: average colony diameter (mm) in the control; *T*: average colony diameter (mm) in the treatment.

### 2.12 Antifungal Analysis on Strawberries

The antifungal activity on strawberries was evaluated using the concentration that showed the highest and statistically significant inhibition of fungal growth in the *in vitro* assays. Fresh strawberries were initially sanitized by immersion in a sodium hypochlorite solution (200 ppm) for 10 min, followed by rinsing with distilled water. After sanitization, the fruits were dried in a laminar flow cabinet for 10 min. Subsequently, each strawberry was individually immersed for 10 s in 50 mL of an aqueous solution containing the inclusion complex dispersed with Tween 80 (0.05%, v/v), at a concentration equivalent to 6400 μM of nerolidol (71.23 mg per 50 mL). In parallel, a control group was prepared under identical conditions, with fruits immersed only in Tween 80 (0.05%, v/v) solution, without the inclusion complex. After treatment, all fruits were dried in a laminar flow cabinet for 30 min to remove excess surface moisture.

Following drying, the strawberries were artificially inoculated by depositing 10 μL of a *B. cinerea* spore suspension (10^6^ spores mL^–1^) onto the fruit surface. The inoculated fruits were kept under a laminar hood for an additional 15 min to allow fungal fixation. The strawberries were then packaged in commercial PET containers (5.5 cm × 10 cm), and stored under refrigerated conditions at 5 °C for 10 days. Three independent containers, with four fruit samples per container, served as the true experimental units (triplicates) for each treatment. Weight loss of the inoculated fruits was determined by recording the mass of all four fruits from each independent container at the beginning and end of storage using a precision balance (BEL Engineering, Monza, Italy), essentially as described elsewhere.^22^ Color stability was analyzed using the CIE Lab* system. Measurements were performed with a portable colorimeter (CR-400; Konica Minolta, Tokyo, Japan), previously calibrated with a white standard, on the surface of all four fruits from each replicated container while avoiding damaged areas. The total color difference (Δ*E*) was calculated according to the standard CIE equation.^22^

### 2.13 Statistical Analysis

All experimental data are expressed as mean ± standard deviation, and the experiments were performed in triplicate (*n* = 3). For the postharvest strawberry assays, each independent container was treated as the true experimental unit (*n* = 3 replicated packages per treatment), with four internal fruit subsamples evaluated per container to capture biological variability (*n* = 12 total measurements per treatment). Statistical analyses were carried out using GraphPad Prism software (version 6.0, GraphPad Software Inc., Boston, MA, USA). Comparisons between groups were performed using two-way analysis of variance (two-way ANOVA), followed by Tukey’s test. For comparisons between two independent groups, an unpaired *t* test with Welch’s correction was applied. Differences were considered statistically significant when *p* < 0.05. For particle size and zeta potential measurements, the data were processed using Kalliope software (version 3.14, Anton Paar), based on six automated instrumental runs per sample to generate a single data point for each independent sample (*n* = 3).

## 3 Results

### 3.1 Physicochemical Characterization of the β-CD/NE Complex

#### 3.1.1 CE %, DLS and Zeta Potential

The β-CD/NE inclusion complex was evaluated by CE %, *Z-*average hydrodynamic diameter, disparity (*D̵*), and zeta potential. The results obtained are summarized in Table 1. It is important to denote that the measured values do not represent the size of a single β-CD/NE host–guest complex, but rather indicate the formation of self-assembled supramolecular aggregates or cluster domains upon redispersion of the lyophilized powder.

**Table 1:** Physicochemical Characteristics of Inclusion Complex Suspensions

sample	CE %	*Z*-average diameter (nm)^b^	peak size (nm)^c^	dispersity (*D̵*)	zeta potential (mV)
β-CD/C	-	245.9 ± 10.9^b^	292.0 ± 136.8^a^	0.250 ± 0.008^a^	–33.0 ± 0.7^a^
β-CD/NE	96.71 ± 0.18	484.9 ± 2.9^a^	386.6 ± 62.3^a^	0.266 ± 0.003^a^	–27.2 ± 1.7^a^

a Values are expressed as mean ± standard deviation (*n* = 3). Different letters indicate statistically significant differences among complexes (*p* < 0.05).

b Intensity-weighted *Z*-average diameter refers to the harmonic intensity-weighted average hydrodynamic diameter determined by the cumulants method according to ISO 22412.

c Peak size represents the mean hydrodynamic diameter of specific particle populations, calculated from the apex of individual peaks in the intensity size distribution using the CONTIN algorithm.

The β-CD/NE exhibited a high complexation efficiency, indicating effective incorporation of NE into the β-CD cavity. Dynamic light scattering measurements revealed particle sizes in the nanometric range for both systems, with a unimodal size distribution and moderate polydispersity. Zeta potential analysis showed negative surface charges for both the control and the β-CD/NE complex, indicating similar electrokinetic behavior in aqueous medium. The corresponding particle size and zeta potential distributions of the formulations are presented in Figure S2.

#### 3.1.2 Electron Microscopy Analysis

SEM micrographs (Figure 2) of the β-CD/NE revealed the presence of irregularly shaped particles forming agglomerated structures. The sample consisted predominantly of clustered particles with heterogeneous sizes and nonspherical morphology. The aggregates were distributed unevenly over the surface, forming compact clusters with a rough and fragmented appearance.

**Figure 2:**
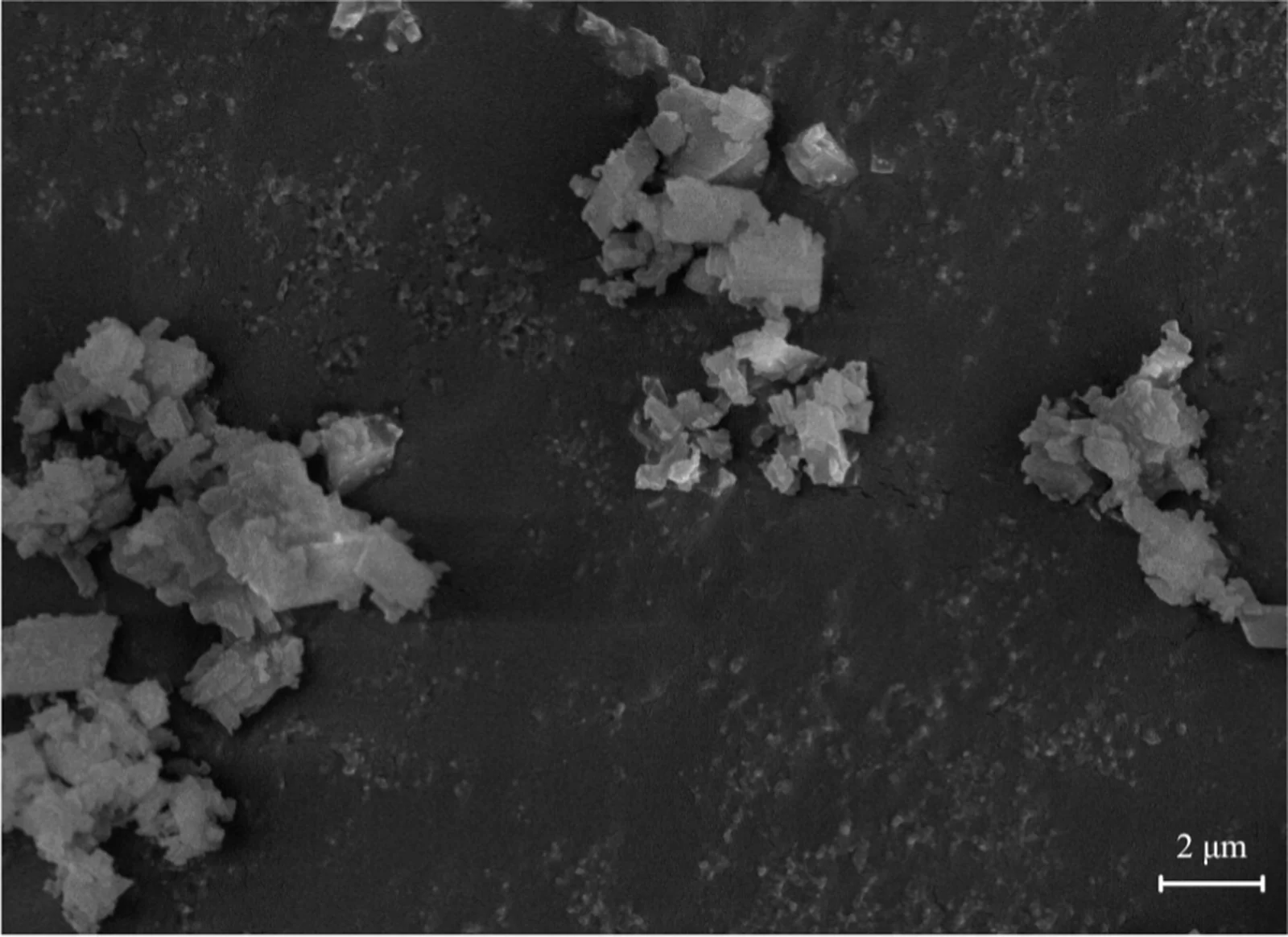
Scanning electron microscopy (SEM) images of β-CD/NE inclusion complex at 10,000× magnification. Bar = 2 μm.

#### 3.1.3 FTIR

The FTIR spectra of NE, β-CD and β-CD/NE are shown in Figure 3A. NE exhibited characteristic absorption bands associated with C–H stretching in the high-wavenumber region (range 2968–2918 cm^–1^), and signals at approximately 1450 and 1376 cm^–1^ corresponding to C–H bending vibrations. Bands at 1108 and 995 cm^–1^ were associated with C–O stretching and =C–H out-of-plane bending modes. The β-CD spectrum showed a broad band around 3400 cm^–1^ assigned to O–H stretching vibrations, typical of hydrogen-bonded hydroxyl groups, and intense absorptions in the carbohydrate region (1200–900 cm^–1^), particularly at 1021 cm^–1^, related to C–O–C and C–O stretching of the glucopyranose units.

**Figure 3:**
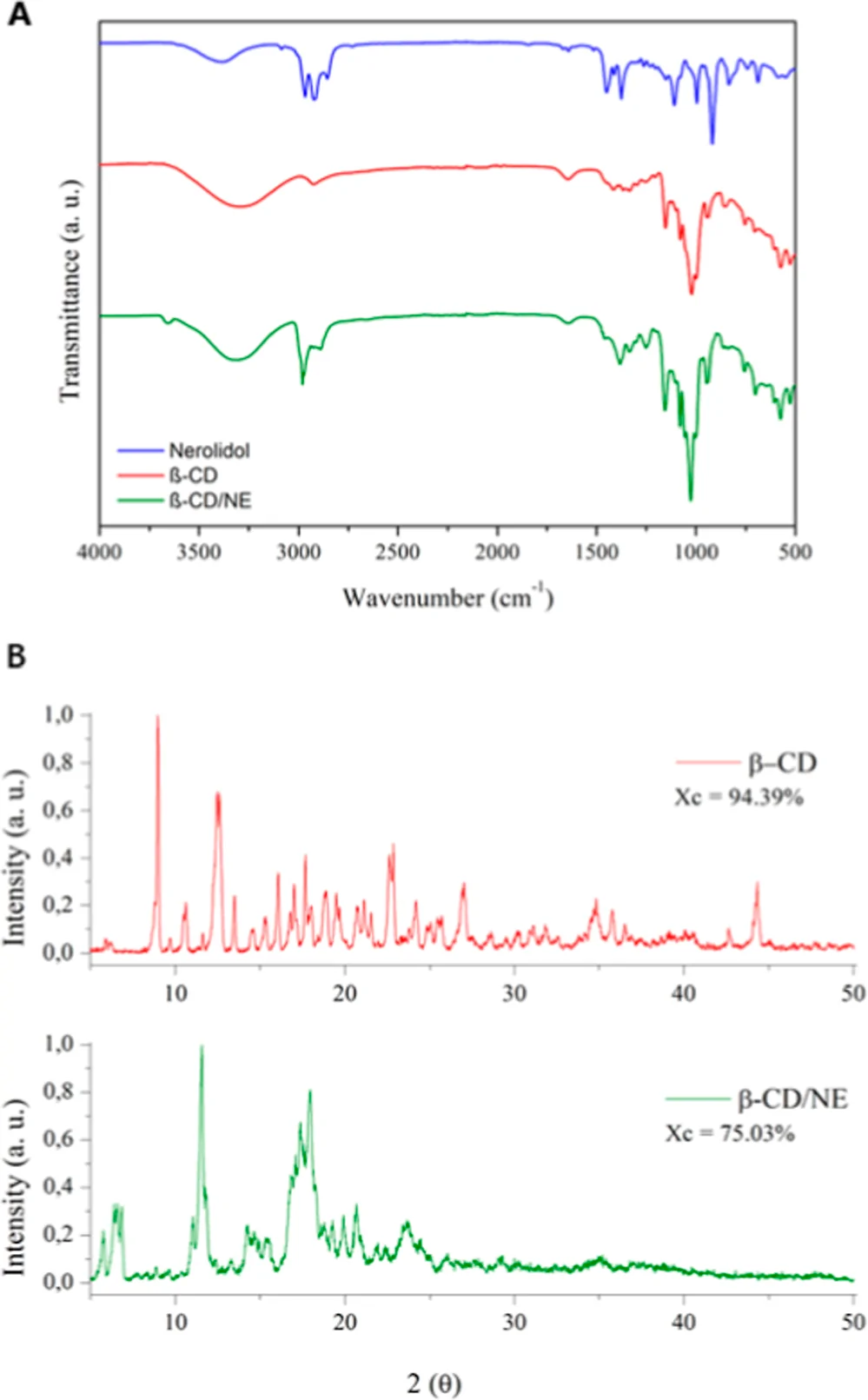
(A) FTIR spectra of nerolidol, β-cyclodextrin (β-CD) and the β-CD/NE. (B) XRD diffractograms of β-CD and the β-CD/NE, showing the corresponding crystallinity indices.

In the β-CD/NE spectrum, bands characteristic of both components were observed. In the 3000–2850 cm^–1^ region, attributed to aliphatic C–H stretching vibrations of NE, changes in band profile and relative intensity were detected compared with free NE, suggesting alterations in the vibrational environment of the guest molecule upon interaction with β-CD. Additionally, modifications in the 1200–900 cm^–1^ region, along with changes in band shape and relative intensity, suggested molecular interaction between NE and β-CD.

#### 3.1.4 XRD

The XRD diffractograms of β-CD and β-CD/NE inclusion complex are shown in Figure 3B. The β-CD exhibited a crystalline diffraction pattern characterized by multiple sharp reflections throughout the angular range, with a crystallinity index (*X*c) of 94.39%. The diffractogram of the β-CD/NE also presented a crystalline profile, with a *X*c of 75.03%. In the β-CD/NE diffractogram, peaks were observed in the same angular region as those of β-CD, although with differences in relative peak intensity and background profile.

#### 3.1.5 SAXS

SAXS curves and structural analysis are presented in Figure 4. The background-subtracted scattering profiles of the β-CD/NE (Figure 4A) exhibited a clear power-law decay in the intermediate *q*-range (0.01–0.1 Å^–1^). Fitting of the data using a power-law model (*I*(*q*) = *Aq* ^–*m*^) in this region (Figure 4B) yielded Porod exponents of 4.0003, 4.0004, and 4.0194 for the three independent replicates. The mean exponent was *m* = 4.007 ± 0.011 (mean ± standard deviation, *n* = 3).

**Figure 4:**
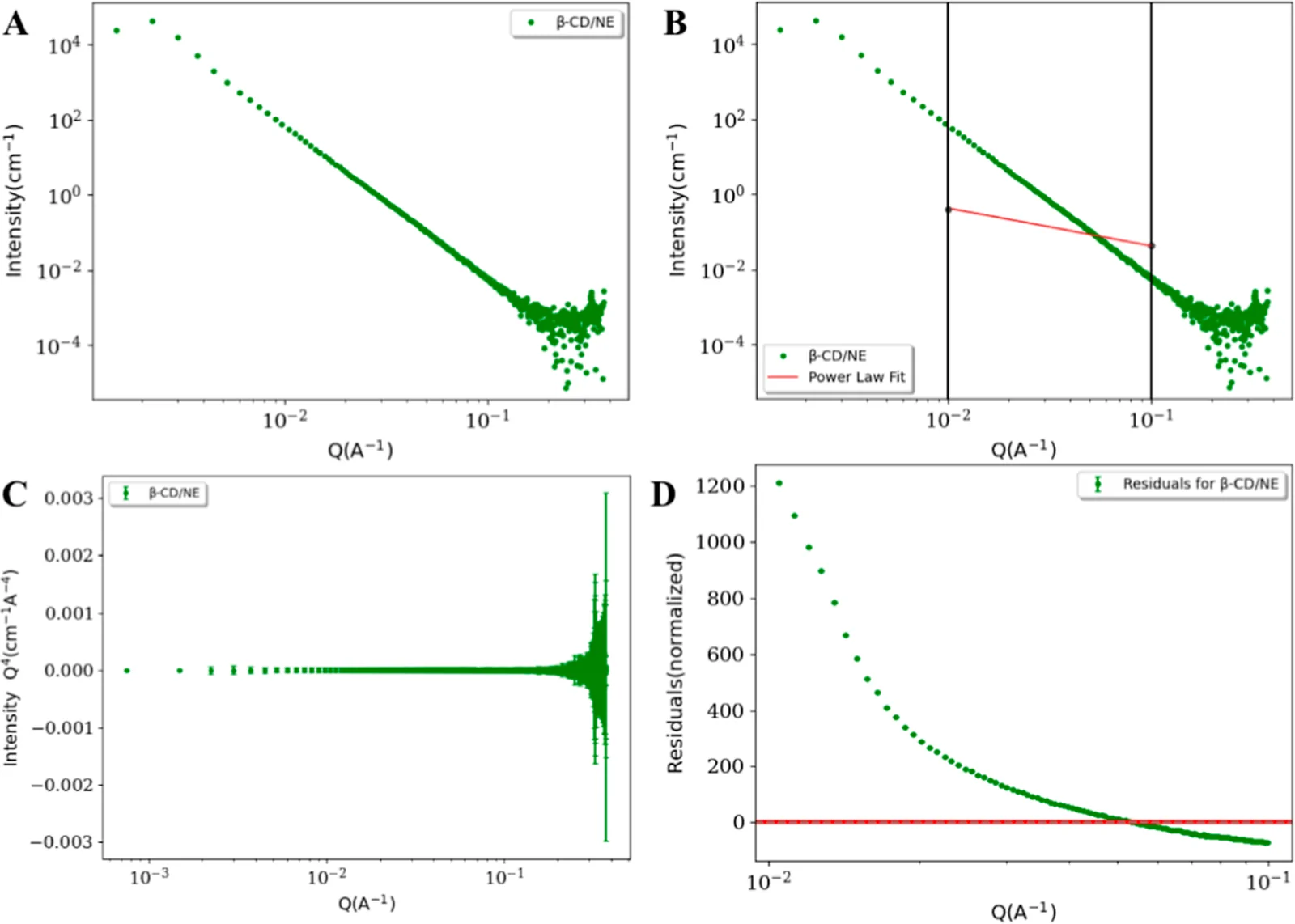
SAXS characterization of the β-CD/NE inclusion complex. (A) Scattering intensity after background subtraction (log–log scale). (B) Power-law fitting in the range 0.01–0.1 Å^–1^. (C) Porod plot (*I*(*q*)*q* ^4^ vs *q*). (D) Normalized residuals of the fitting procedure.

The corresponding surface fractal dimension, calculated as *D*_s_ = 6 – *m*, was 1.993 ± 0.011. The Porod prefactors (*A*) were on the order of 10^–11^ for all samples, with minor variations among replicates. The Porod plot (*I*(*q*)·*q* ^4^ vs *q*) is shown in Figure 4C, and the normalized residuals of the power-law fitting are presented in Figure 4D.

#### 3.1.6 ^13^C NMR

The solid-state ^13^C NMR spectra of native β-CD and β-CD/NE complex are presented in Figure 5. The spectrum of β-CD exhibited its characteristic resonances at 97.73, 76.35, 67.60, and 56.43 ppm, together with less intense signals distributed between 20 and 100 ppm. After complexation, the β-CD/NE spectrum showed resonances at 97.71, 74.87, 66.61, and 54.71 ppm, accompanied by additional signals at 139.24, 129.13, 128.43, and 106.69 ppm. Variations in signal positions and intensities were observed when compared with native β-CD.

**Figure 5:**
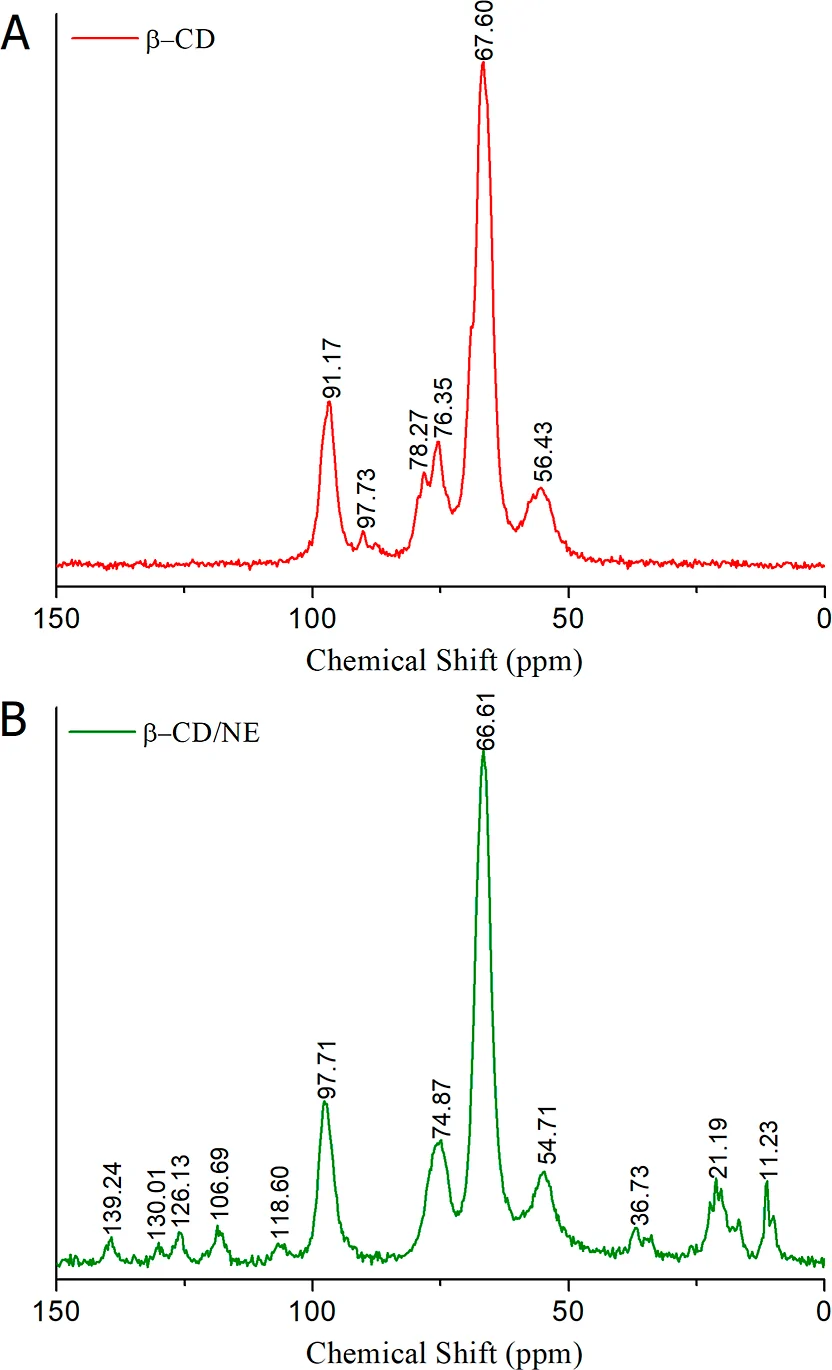
Solid-state ^13^C CP/MAS NMR spectra of (A) native β-cyclodextrin (β-CD) and (B) β-cyclodextrin/nerolidol inclusion complex (β-CD/NE).

In addition, the spectrum of the β-CD/NE complex displayed resonances in the region between approximately 106 and 139 ppm, whereas β-CD showed no prominent signals in this range. Signals associated with the β-CD structure remained present in both spectra, although slight changes in their chemical shifts were detected following complex formation.

#### 3.1.7 Thermal Analysis

The DSC thermograms of β-CD, NE and β-CD/NE are shown in Figure 6. The β-CD sample (Figure 6A) presented a single endothermic event with a peak temperature of 117.26 °C and an associated enthalpy (Δ*H*) of 403 J g^–1^. The thermogram of NE (Figure 6B) exhibited three thermal events, with endothermic peaks at 130.50 °C (Δ*H* = −0.57 J g^–1^), 145.39 °C (Δ*H* = 12.03 J g^–1^) and 213.33 °C (Δ*H* = −8.56 J g^–1^). For the β-CD/NE inclusion complex (Figure 6C), a single thermal event was observed, with a peak temperature of 101.05 °C and enthalpy of 9.97 J g^–1^.

**Figure 6:**
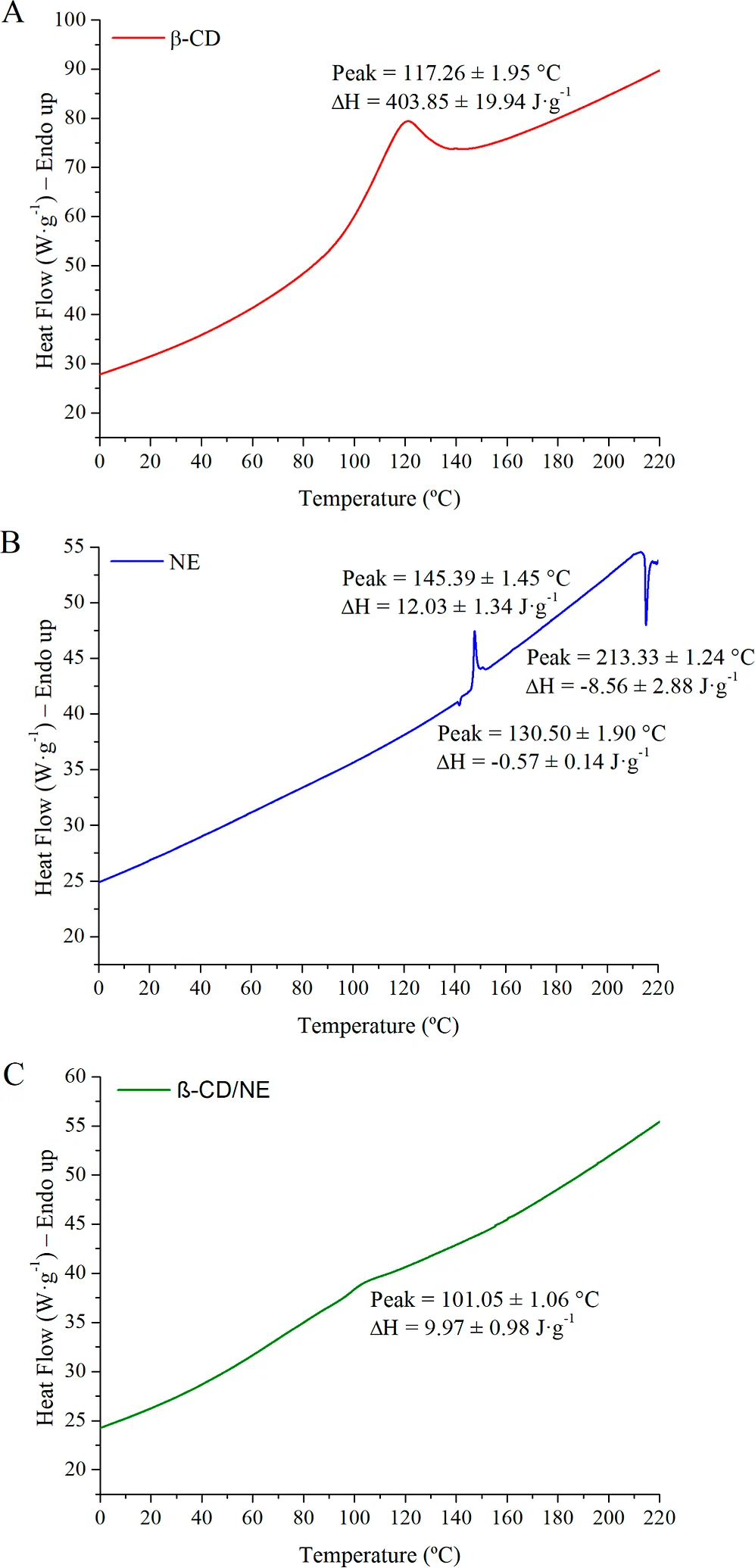
DSC thermograms of (A) β-CD, (B) NE, and (C) the β-CD/NE. Values are expressed as mean ± standard deviation (*n* = 3).

The TGA and DTG curves of β-CD, NE and β-CD/NE are shown in Figure S3. The β-CD sample exhibited an initial mass loss at low temperatures, followed by a major decomposition stage occurring in a single event in the range of approximately 300–400 °C, as confirmed by the DTG peak, and with a residual mass remaining at the end of the analysis. NE showed a different thermal profile, characterized by a single and intense mass loss event occurring at lower temperatures, with almost complete volatilization of the sample and negligible residue at high temperatures, as evidenced by the sharp DTG peak. For the β-CD/NE, the TGA curve showed an initial gradual mass loss at low temperatures, followed by a main decomposition event in the same temperature region observed for β-CD, which was also reflected in the DTG curve. A residual mass was also observed at the end of the heating program.

### 3.2 Antifungal Activity *In Vitro*

Visual inspection of the agar plates confirmed a progressive reduction in mycelial growth for both treatments, with greater inhibition observed for β-CD/NE at the same concentrations (Figure 7A). The β-CD/NE complex and NE alone inhibited the mycelial growth of *B. cinerea* in a concentration-dependent manner (Figure 7B). At 200 μM, β-CD/NE promoted an inhibition percentage (IP) of 17.9 ± 5.2%, whereas NE exhibited minimal activity (0.55 ± 0.49%). A gradual increase in antifungal effect was observed with increasing concentrations of β-CD/NE, reaching 28.8 ± 5.3%, 33.1 ± 2.2% and 43.8 ± 1.8% at 400, 800, and 1600 μM, respectively. At higher concentrations, β-CD/NE achieved IP values of 52.4 ± 4.2% at 3200 μM and 65.8 ± 1.5% at 6400 μM.

**Figure 7:**
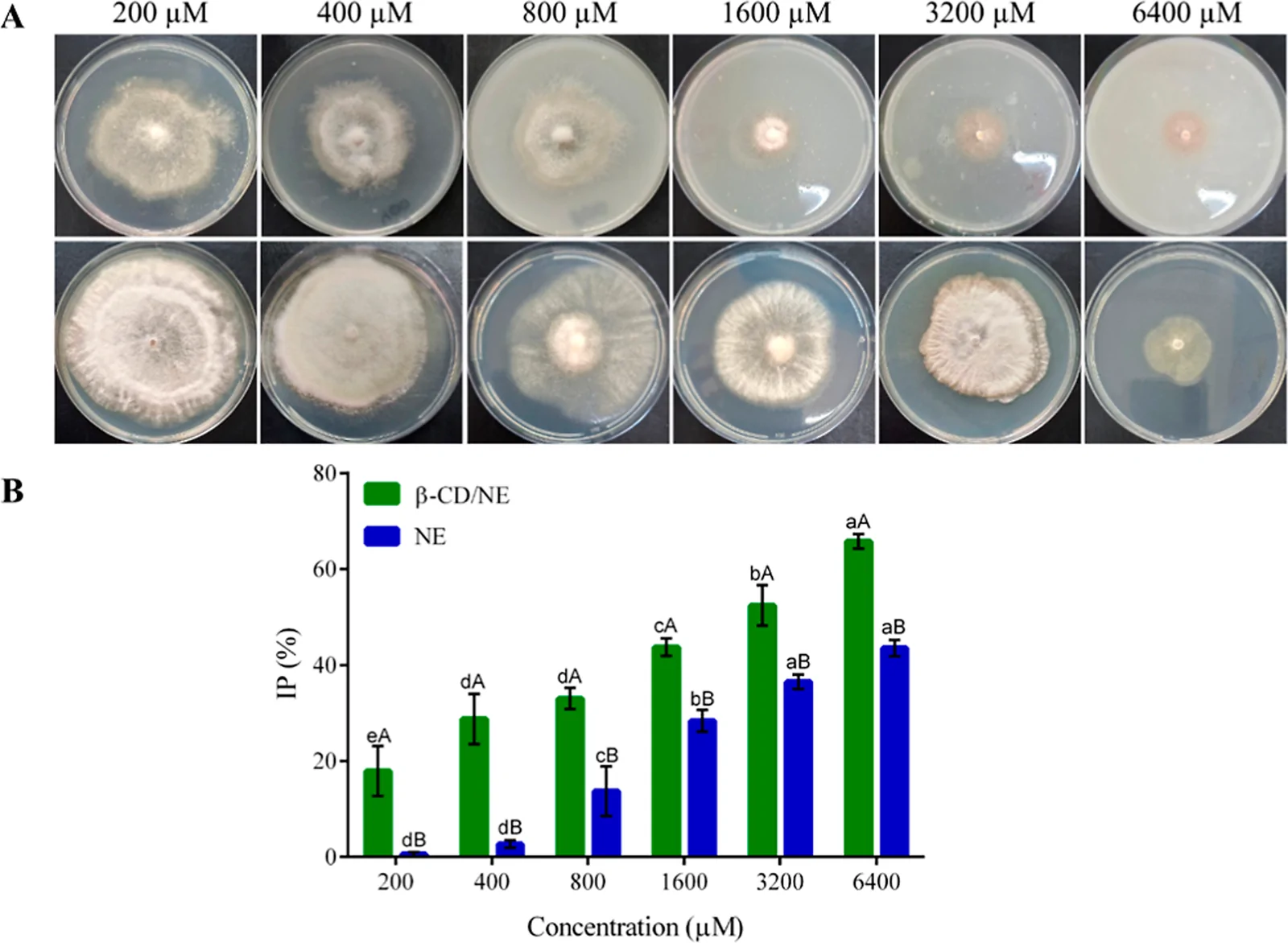
(A) Antifungal activity of β-CD/NE (top row) and NE (bottom row) against *Botrytis cinerea* at different concentrations. (B) Inhibition percentage of β-CD/NE and NE against *Botrytis cinerea* at different concentrations. Values are expressed as mean ± standard deviation (*n* = 3). Lowercase letters indicate significant differences between concentrations within the same treatment, uppercase letters indicate differences between treatments at the same concentration (two-way ANOVA followed by Tukey’s test, *p* < 0.05).

For the NE treatment, inhibition also increased as a function of concentration, with IP values of 2.73 ± 0.83% (400 μM), 13.7 ± 5.2% (800 μM), 28.4 ± 2.3% (1600 μM), 36.6 ± 1.5% (3200 μM) and 43.6 ± 1.7% (6400 μM). Significant differences (*p* < 0.05) were detected both between concentrations within each treatment and between β-CD/NE and NE at the same concentration.

### 3.3 Antifungal Activity in Strawberry Fruits

The visual appearance of strawberries at day 0 and after 10 days of refrigerated storage following inoculation with *B. cinerea* is depicted in Figure 8. At the initial time (day 0), both the control fruits (Figure 8A) and those treated with the β-CD/NE complex (Figure 8C) exhibited uniform coloration and healthy visual appearance, without signs of fungal contamination. After 10 days of storage, pronounced fungal development was observed in the control group (Figure 8B). All fruits presented visible fungal contamination, characterized by surface darkening and tissue deterioration, with one strawberry displaying intense mycelial mass covering a large portion of the fruit surface. In contrast, no visible fungal growth was detected on the strawberries treated with the β-CD/NE complex after 10 days (Figure 8D). However, subtle changes in fruit appearance were observed, including a loss of surface brightness and a slight reduction in color intensity, particularly in one of the samples.

**Figure 8:**
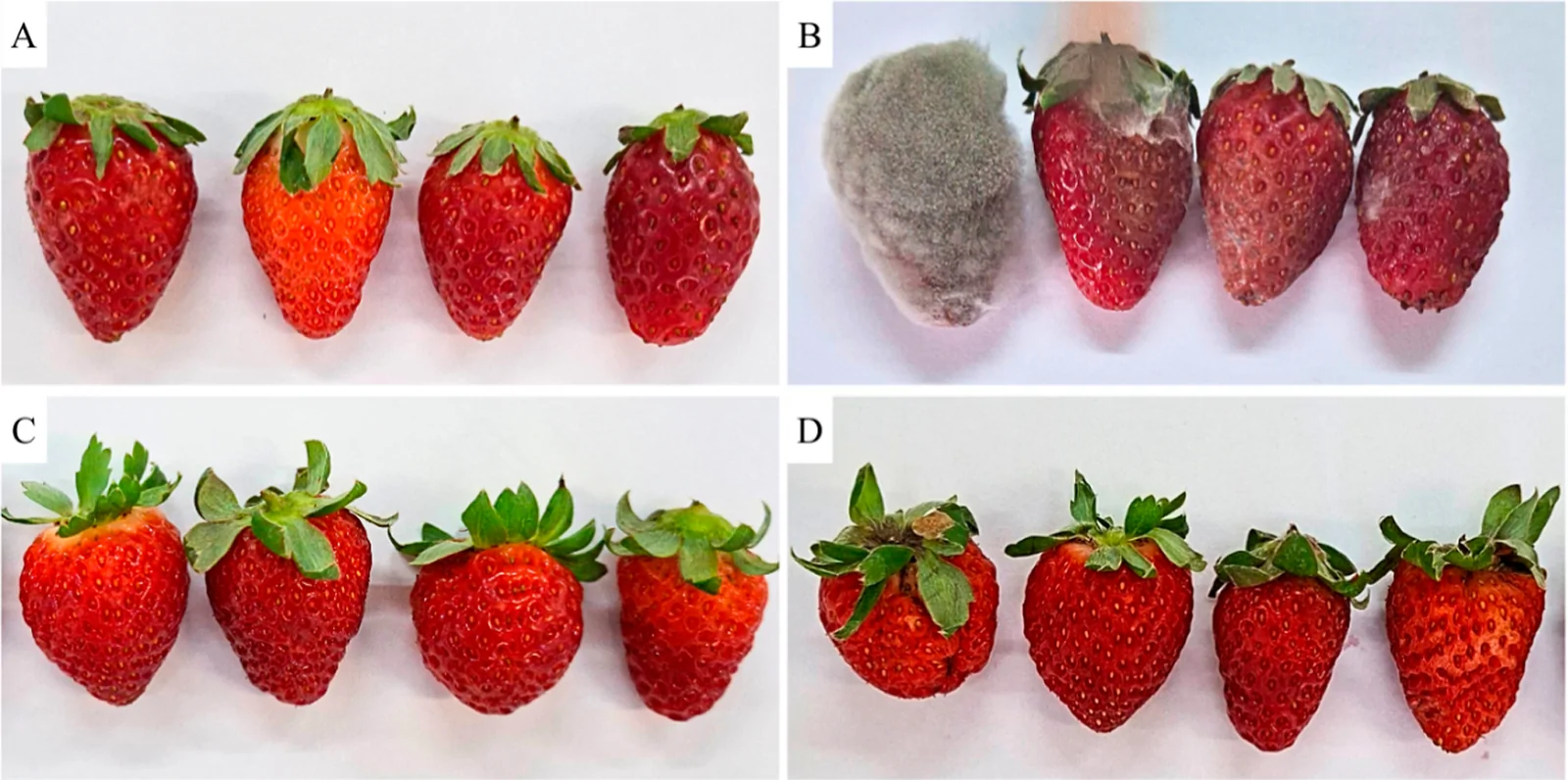
Visual appearance of strawberries inoculated with *Botrytis cinerea* and stored at 5 °C for 10 days. (A,B) Control fruits; (C,D) fruits treated with the β-CD/NE complex. Panels (A,C) correspond to day 0, and panels (B,D) to day 10 of storage.

The control group showed a mean weight loss of 19.9 ± 4.4%, while fruits treated with β-CD/NE lost only 8.7 ± 2.7% of their initial mass. This difference was statistically significant according to an unpaired *t* test with Welch’s correction (*p* = 0.0285), indicating that the treatment effectively limited mass loss during storage. A similar trend was observed for color stability. Control fruits presented a Δ*E* value of 17.21 ± 1.27, reflecting pronounced color changes, whereas treated samples exhibited a lower Δ*E* of 6.71 ± 0.38. The difference between treatments was highly significant (Welch’s correction, *p* = 0.0027), confirming that β-CD/NE markedly preserved the visual quality of the strawberries over the storage period.

## 4 Discussion

The β-CD/NE inclusion complex exhibited a high CE together with nanoscale hydrodynamic diameters and low polydispersity values, indicating the formation of well-defined colloidal structures. The β-CD/NE complex showed a CE > 96%, indicating that almost the entire NE fraction was successfully retained within the β-CD matrix. This value was higher than that described in a previous study using the same preparation conditions (1:1 molar ratio, 400 rpm stirring for 36 h, followed by freeze-drying), where a CE of 70% was achieved.^11^ The authors attributed the lower recovery to volatilization losses during processing rather than to limitations of the host–guest interaction. In our study, the CE results indicate that the complexation and drying conditions were particularly effective in preventing terpene evaporation and favored its stabilization inside the β-CD cavity. A comparable CE of 90% has been reported in systems employing modified cyclodextrins (HP-β-CD) in nanofibers,^20^ rather than conventional β-CD aqueous complexes, thereby reinforcing the robustness of the encapsulation strategy adopted here.

The morphological features observed by SEM are in agreement with these physicochemical findings. The β-CD/NE complex displayed compact aggregates with irregular surfaces and reduced crystal domains, a pattern similar to that previously described for β-CD/NE inclusion complexes, where the loss of the native crystalline habit of β-CD was interpreted as evidence of successful guest–host inter action.^11^ In contrast to the well-defined cubic/prismatic crystals reported for the β-CD/4-terpineol complex,^23^ the β-CD/NE system exhibited irregular aggregates with reduced crystalline features, suggesting a distinct structural reorganization likely influenced by the preparation method. Compared with the structures reported for HP-β-CD/NE systems, which exhibited more diffuse and poorly defined lyophilized aggregates,^19^ the morphology obtained in the present work appears more organized and consistent with the formation of a stable inclusion complex rather than a simple physical mixture.

The FTIR and XRD analyses provide evidence of NE encapsulation within the β-CD cavity. In the FTIR spectra, the main absorption bands of NE were attenuated in the β-CD/NE complex, whereas the spectral profile of β-CD remained predominant. This masking effect has been interpreted as an indicator of molecular inclusion, since encapsulated guests exhibit restricted vibrational mobility and reduced infrared absorption.^11,17,24^ A comparable attenuation pattern was reported for thymol-β-CD and carvacrol-β-CD systems, where guest vibrational bands were either weakened or shifted after complexation.^4,25^ The disappearance of typical 4-terpineol bands and O–H band shifts were observed after complexation with β-CD.^23^ In contrast, the β-CD/NE complex showed modifications mainly in the C–H stretching and fingerprint regions, without pronounced O–H shifts, indicating a distinct interaction pattern. The XRD results support this interpretation, while pure β-CD showed a highly crystalline diffraction pattern, the β-CD/NE complex exhibited broader and less intense peaks with increased amorphous contribution. The formation of stable β-CD host–guest hydration crystals requires a meticulous balance between hydrophobic attraction and an optimized, matching hydrogen-bond network within the solid-state lattice.^26,27^ The decrease in the crystallinity index observed for β-CD/NE (75.03%) relative to native β-CD (84.39%) corroborates this structural rearrangement. The guest encapsulation and its subsequent interactions effectively disrupt the highly ordered long-range packing typically found in native cyclodextrin structures, leading to a higher amorphous character.^11,26,27^

The SAXS profiles of the β-CD/NE exhibited a well-defined power-law decay in the intermediate *q*-range, with Porod exponents of 4.007 ± 0.011, indicating that the system reaches the classical Porod regime characteristic of two-phase systems with sharp electron density contrast and smooth interfaces.^26,27^ This asymptotic Porod behavior, and a surface fractal dimension of 1.993 ± 0.011, mathematically rules out randomized or amorphous physical aggregation. Instead, it supports a highly organized, compact nanoscale arrangement with well-defined domain boundaries.^26,28^ The Porod representation (*I*(*q*)·*q* ^4^ vs *q*) displayed an approximately constant region within the fitting window, corroborating the establishment of asymptotic Porod behavior and well-defined domain boundaries, with deviations at high *q* attributed to reduced signal-to-noise ratio, as commonly reported for carbohydrate-based supramolecular systems.^28^ When interpreted alongside XRD results, the SAXS data indicate that inclusion disrupts long-range crystalline packing while preserving compact nanoscale domains rather than generating amorphous or fractal aggregates. Similar structural behavior, characterized by decreased crystallinity combined with preserved nanoscale organization, has been reported for cyclodextrin-based inclusion systems.^29^ Collectively, the SAXS, XRD and FTIR results demonstrate that NE incorporation into β-CD leads to a dense supramolecular assembly characterized by sharp internal interfaces, reduced long-range order and high nanoscale structural reproducibility.

The appearance of additional resonances in the β-CD/NE spectrum is consistent with the incorporation of NE into the cyclodextrin system. Previous ^13^C NMR studies have reported characteristic NE signals at approximately 145 ppm (C2), 135 ppm (C7), 131 ppm (C11), 124 ppm (C6 and C10), 111 ppm (C1), and 73 ppm (C3).^30,31^ In the present study, new resonances were observed at 139.24, 129.13, 128.43, and 106.69 ppm in the β-CD/NE spectrum, which fall within the chemical shift range reported for the olefinic carbons of nerolidol, particularly those assigned to C1, C2, C7, and C11.^30,31^ It should be noted that slight differences in chemical shift values are expected due to the distinct experimental conditions employed for solution-state NMR analyses reported in the literature and the solid-state ^13^C CP/MAS NMR technique used in the present study. In addition, slight changes were detected in the characteristic β-CD resonances after complexation, including shifts from 76.35 to 74.87 ppm and from 67.60 to 66.61 ppm. Such variations in chemical shift indicate modifications in the electronic environment of the cyclodextrin carbons, which may be attributed to interactions between the guest molecule and the β-CD cavity. These cross-polarization solid-state shifts provide direct evidence of localized host–guest interactions inside the hydrophobic cavity, a phenomenon that would not occur in a simple physical mixture where both components retain their native, unaltered chemical shifts.^30^ Similar alterations in NMR profiles have been reported for terpene/β-CD inclusion complexes, where host–guest interactions lead to changes in the chemical environment of both components.^32^

Thermal analysis further supported the occurrence of molecular encapsulation and indicated that NE became thermally stabilized after complexation with β-CD. In the DSC curves, free NE exhibited distinct evaporation- and degradation-related events, whereas these transitions were markedly attenuated in the β-CD/NE complex, which presented a smoother thermal profile. Similar reduction and broadening of thermal events have been reported for terpene-β-CD inclusion systems, where the disappearance or displacement of guest-related peaks is attributed to reduced volatility and restricted molecular mobility inside the β-CD cavity.^4,11^ The TGA/DTG curves also followed the expected behavior for inclusion complexes: while NE underwent an early mass loss associated with volatilization, the β-CD/NE complex showed a delayed degradation pattern, with mass loss occurring predominantly in the same temperature region as β-CD. This shift toward the decomposition range of the host material has been widely interpreted as evidence that volatile guests are retained within the β-CD matrix and are released only upon carbohydrate degradation.^11,14^ These results corroborate the structural analyses, suggesting that NE is entrapped within the β-CD cavity and that complexation reduces its volatility, a feature that is particularly relevant for its subsequent antifungal application.

The significantly higher IP % observed for the β-CD/NE complex compared to free NE suggests that encapsulation in β-CD overcomes the inherent limitations of pure NE. As a sesquiterpene, NE is characterized by high hydrophobicity and high volatility,^33^ which limits its bioavailability and persistence in the aqueous environment of the agar medium. Thus, the formation of inclusion complexes enhances the aqueous solubility of hydrophobic guests by shielding the nonpolar moieties within the β-CD cavity.^19^ This likely facilitated a more uniform distribution of NE in the culture medium, leading to the more pronounced reduction in *B. cinerea* mycelial growth observed in the present study.

Furthermore, the concentration-dependent response observed here is consistent with the findings reported for other essential oil constituents.^17^ In this context, the controlled-release behavior of β-CD plays a key role. While free NE is likely to volatilize rapidly during the incubation period, its complexation with β-CD allows the system to function as a reservoir, sustaining an effective concentration of the bioactive compound in close contact with the fungal hyphae for an extended period.^17,33^ In addition, the partial amorphization of the β-CD crystalline lattice upon guest incorporation, as evidenced by the XRD results discussed previously, supports the existence of strong host–guest interactions that contribute to the stabilization of the bioactive molecule.^4,13^

The complete inhibition of mycelial growth in treated strawberries underscores the potential of this technology to mitigate food waste. According to predictive studies on strawberry losses, *B. cinerea* is the primary driver of commercial value degradation, and its rapid proliferation under storage conditions necessitates effective control strategies.^5^ In this context, the successful performance of the β-CD/NE treatment under refrigerated storage (5 °C) aligns with current trends in active packaging for berries, in which the maintenance of a modified microatmosphere enriched with bioactive vapors is considered necessary for extended preservation.^34^ The preservation observed in treated fruits can therefore be attributed to the sustained release of NE vapors from the inclusion complex, which establishes an antifungal microenvironment at the fruit surface.^35,36^

This prolonged postharvest efficacy highlights the critical impact of supramolecular encapsulation. While free volatile sesquiterpenes typically undergo rapid evaporation and nonuniform surface distribution when directly applied to fresh produce, the host–guest inclusion within β-CD cavity acts as a protective shield.^13,19^ By significantly reducing the chemical volatility and preventing early terpene loss, the β-CD/NE ensures that a sustainable, effective dose of bioactive vapors is continuously available to suppress *B. cinerea* germination and mycelial proliferation throughout the extended storage period, a protective performance that cannot be replicated by the highly volatile free compound alone.^17,19^ Although the *in situ* results suggest that the β-CD/NE system can successfully protect the fruit produce, further specific diffusion and shelf life control studies are required to fully discriminate the quantitative enhancement brought by molecular complexation on the strawberry surface.

The reduction in mass loss from 19.9% in the control group to 8.7% in treated fruits represents a relevant outcome from an industrial perspective. Once, treatments based on active compounds can reduce fruit respiration rate and help maintain cellular membrane integrity, thereby delaying water loss and endogenous sugar consumption.^5,36,37^ Furthermore, considering that color is one of the main attributes associated with consumer acceptance, especially in strawberries, whose red hue is related to the presence of anthocyanins and the state of ripeness, the lower Δ*E* in the treated samples suggests less pigment degradation during storage.^38^ In addition, the β-CD/NE complex may function as a partial physical barrier to water vapor diffusion at the fruit surface, and more efficient maintenance of surface integrity and the compounds responsible for coloration, a protective mechanism analogous to that reported for bioactive polymeric systems.^20,39^

Thus, this study advances the application of cyclodextrin-based inclusion systems for postharvest disease control by demonstrating the effectiveness of a β-CD/NE inclusion complex specifically developed and evaluated for the control of *B. cinerea* in fresh fruit. While previous studies have reported the encapsulation of NE or other terpenes in cyclodextrins for improving physicochemical stability, solubility, or controlled release in pharmaceutical, cosmetic, or model systems,^10,17,18^ their antifungal performance in real food matrices has remained largely unexplored. In contrast, the present work combines physicochemical characterization with both *in vitro* and *in situ* antifungal assays, providing direct evidence that β-CD complexation enhances the practical efficacy of NE against *B. cinerea* during refrigerated storage of strawberries. The strong CE %, structural modification, and thermal stabilization observed here support the improved biological performance of the encapsulated NE, aligning with stabilization trends expected for CD-terpene systems.^13,14^

## 5 Conclusion

This study reports the successful preparation of a β-CD inclusion complex containing NE (β-CD/NE) by freeze-drying. The system exhibited high complexation efficiency and nanoscale particles with a monomodal size distribution. While FTIR, XRD, and thermal analyses demonstrated strong molecular interactions, matrix lattice disruption, and increased thermal stability, SAXS and ^13^C NMR analyses indicates the host–guest inclusion complex formation through sharp internal interfaces and shifted electronic environments inside the carbohydrate cavity. From a biological perspective, the β-CD/NE overcame the limited solubility and high volatility of free NE, resulting in enhanced antifungal activity against *B. cinerea* *in vitro*. The performance of the system suggested a solid potential under *in situ* conditions, where treatment of strawberries stored at 5 °C led to the suppression of visible fungal growth for 10 days and a marked reduction in fruit mass loss. Overall, these results support the potential use of the β-CD/NE as a food-compatible approach for extending the shelf life of highly perishable fruits, contributing to the practical application of cyclodextrin-based delivery systems in postharvest disease control.

## Supplementary Material



## Data Availability

Data will be made available on request.

## Notes

Representative HPLC chromatograms of nerolidol standard and nonencapsulated fraction; dynamic light scattering (DLS) particle size and zeta potential distribution curves; thermogravimetric analysis (TGA) and derivative thermogravimetry (DTG) curves of precursors and inclusion complex (PDF)
