# Supplementary material for: β‑Cyclodextrin/Nerolidol Inclusion Complex as a Potential Ecofriendly Antifungal System against Botrytis cinerea in Postharvest Strawberries
Source: ACS Omega. 2026 Sep 26;11(39):59828–39. doi: 10.1021/acsomega.6c06824 (PMC13647828; doi:10.1021/acsomega.6c06824)
Supplement: Supplementary file 1 [file ao6c06824_si_001.pdf]

ACS Omega

Supporting Information for:

β-Cyclodextrin/nerolidol inclusion complex as a potential ecofriendly antifungal system against *Botrytis cinerea* in postharvest strawberries

Aldrey N. R. Corrêa[1], Lara E. Zanotelli[1], Jessica F. Hoffmann[2], Adriano Brandelli[1,3,]*

[1] Department of Food Science, Universidade Federal do Rio Grande do Sul, 91501-970 Porto Alegre, Brazil

[2] Technological Institute in Food for Health, Universidade do Vale do Rio dos Sinos, 93022-750 São Leopoldo, Brazil

[3] Center of Nanoscience and Nanotechnology, Universidade Federal do Rio Grande do Sul, 91501-970 Porto Alegre, Brazil

* **Corresponding author**: A. Brandelli, e-mail: abrand@ufrgs.br

Supplementary Figure S1

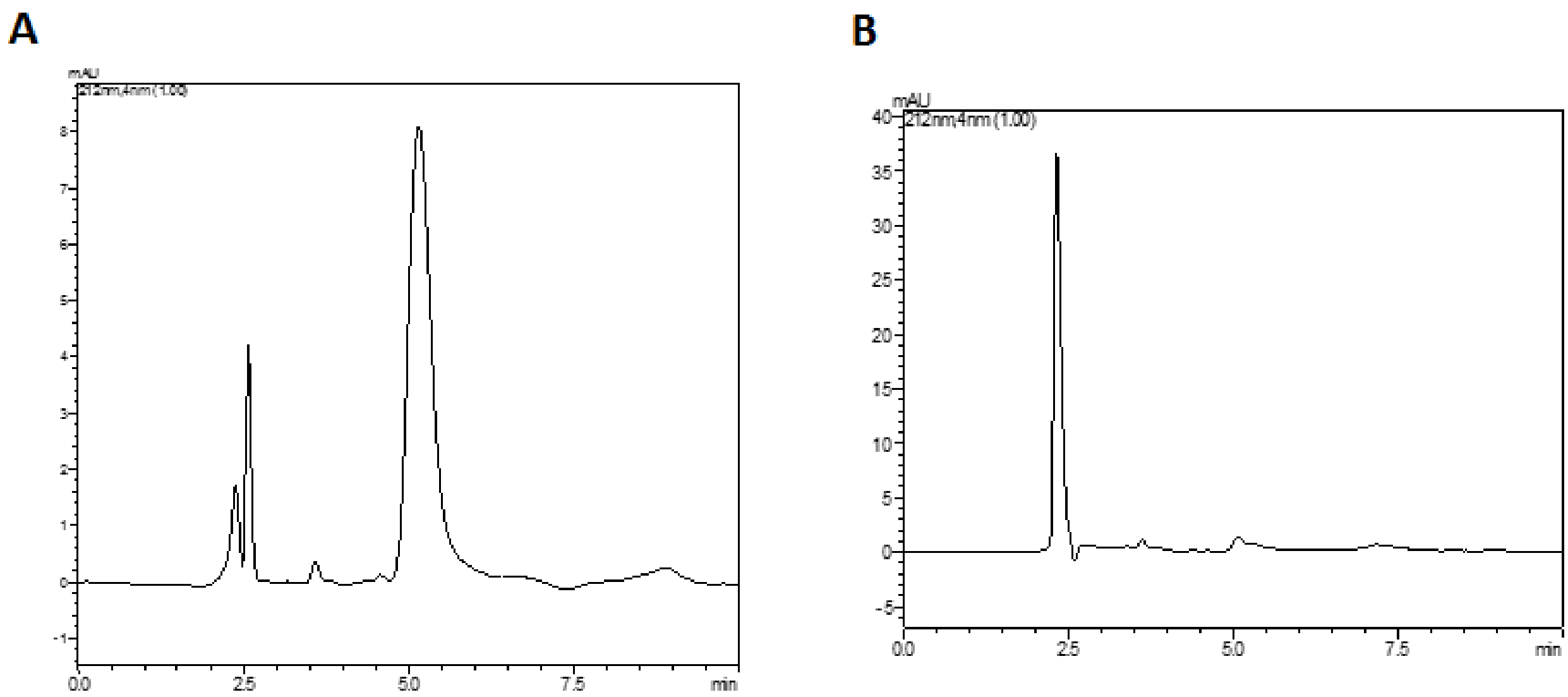

Figure S1. Representative chromatograms of nerolidol. (A) Standard at 100 µg mL^{-1} and (B) sample after ultrafiltration of β-CD/NE complex (filtrate corresponding to non-encapsulated nerolidol).

Supplementary Figure S2

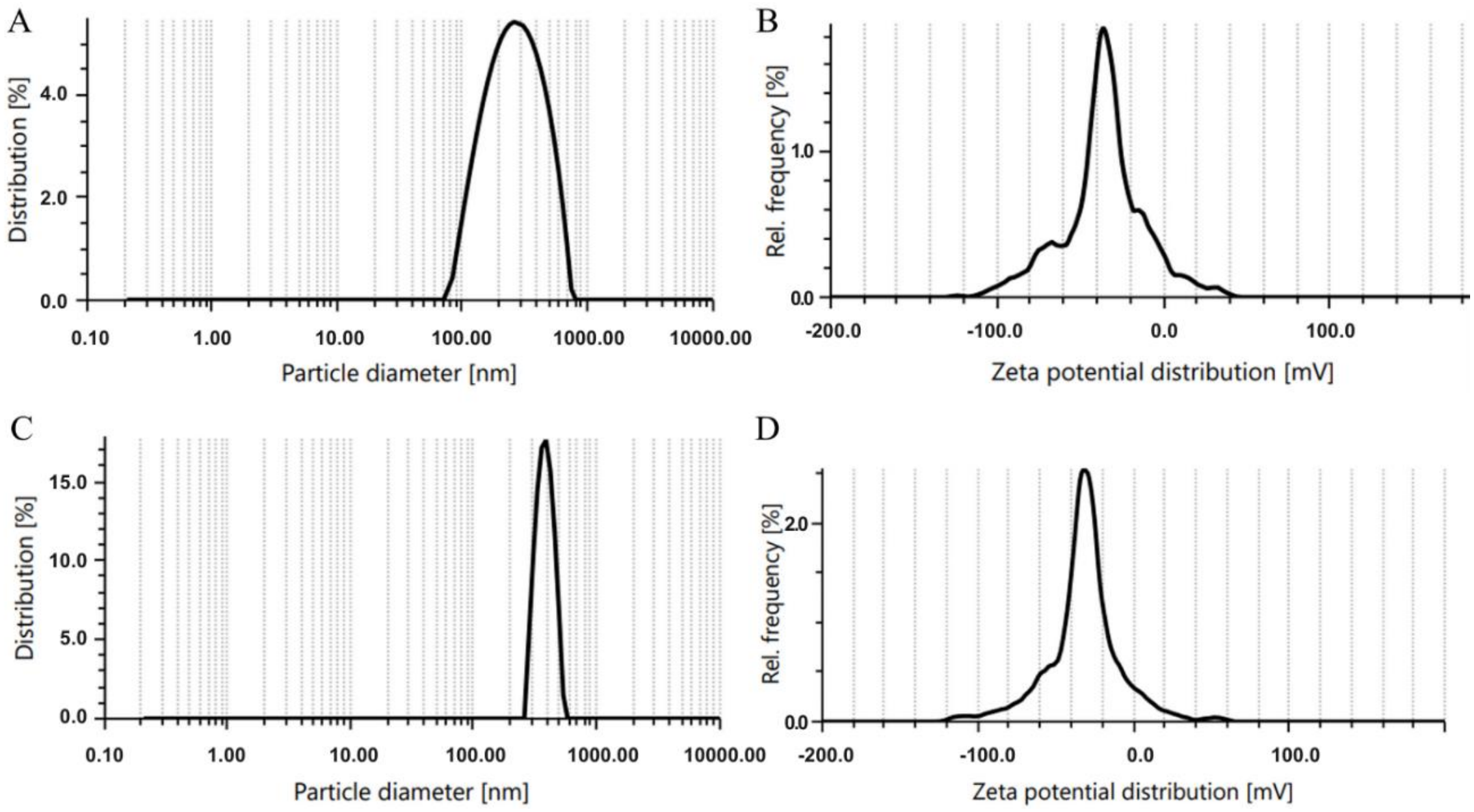

Figure S2. DLS particle size and zeta potential distributions of the control system and the β-CD/NE inclusion complex: (A) DLS and (B) zeta potential of the control (β-CD/C); (C) DLS and (D) zeta potential of the β-CD/NE complex.

Supplementary Figure S3

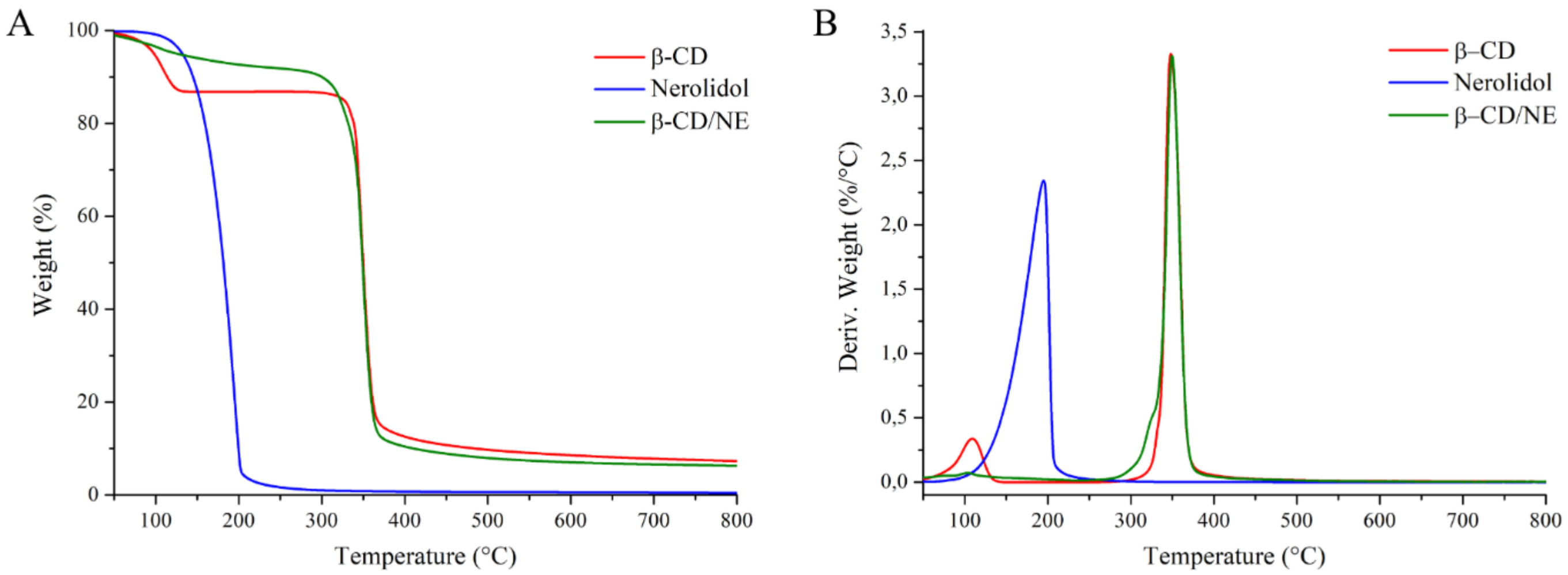

Figure S3. (A) Thermogravimetric analysis (TGA) and (B) first derivative of TGA (DTG) of β-CD, NE and the β-CD/NE.
